# Retraction: Human Cystathionine-β-Synthase Phosphorylation on Serine227 Modulates Hydrogen Sulfide Production in Human Urothelium

**DOI:** 10.1371/journal.pone.0222416

**Published:** 2019-09-12

**Authors:** The PLOS ONE Editors

After publication of this article [1], the following concerns were raised about similarities within western blot images:

- Fig 5C anti-pS/T (top panel), bands in lanes 3 and 5 appear similar to each other;
- Fig 5C anti-HA (bottom panel) bands in lanes 1–4 appear similar to bands in lanes 5–8

The authors cooperated with the editorial follow up by providing the western blot images underlying Fig 5C, along with replicate blots for Fig 5C. The corresponding author has stated that the height and width of the image panels were reset during figure preparation, resulting in a horizontally stretched appearance of the bands. An editorial evaluation of the provided images, to the extent allowed by the image resolution, could not fully resolve the concerns about the images in Fig 5C.

In light of the unresolved concerns about the western blot panels of Fig 5C, the *PLOS ONE* Editors retract this article.

REVB, EM, DE, AR, FF, GC, GR, and RS did not agree with retraction. ED, AI, and VM did not respond.
